# Does physiological tolerance to acute hypoxia and salinity change explain ecological niche in two intertidal crab species?

**DOI:** 10.1093/conphys/coz086

**Published:** 2019-11-28

**Authors:** Thomas R L Falconer, Islay D Marsden, Jonathan V Hill, Chris N Glover

**Affiliations:** 1 School of Biological Sciences, University of Canterbury, Private Bag 4800, Christchurch 8140, New Zealand; 2 Faculty of Science and Technology and Athabasca River Basin Research Institute, Athabasca University, 1 University Dr. Athabasca, Alberta T9S 3A3, Canada; 3 Department of Biological Sciences, University of Alberta, 11445 Saskatchewan Dr. Edmonton, Alberta T6G 2E9, Canada

## Abstract

Intertidal biota is subjected to significant fluctuations in environmental parameters such as salinity and dissolved oxygen (DO). In the current study, the effects of salinity and DO on metabolic rate, critical oxygen partial pressure (*P*_crit_), heart rate and osmoregulation in two intertidal crab species commonly found on New Zealand coastlines, *Hemigrapsus crenulatus* and *Hemigrapsus sexdentatus*, were measured. Based on its habitation of burrows in the lower intertidal zone, *H. crenulatus* was predicted to be more resilient to these environmental stressors than *H. sexdentatus*, which is distributed in the mid to high tidal zone. However, relative to the full-strength seawater control, there were no consistent salinity-dependent changes in respiratory or cardiovascular endpoints in either species following acute 6-h exposures mimicking a tidal cycle. Analysis of haemolymph osmolality and ions determined that both crab species were strong osmotic and ionic regulators over the 6-h exposure period. However, the threshold salinities at which significant changes in osmotic and ionic regulation occurred did differ and generally indicated that *H. crenulatus* was the better regulator. Respiratory and cardiovascular responses to DO were prominent, with a strong bradycardia observed in both species. Changes in osmolality and sodium ion regulation were also seen as DO declined. The effect on sodium ion levels had its onset at a higher oxygen partial pressure in *H. sexdentatus* than in *H. crenulatus*, indicative of a relatively poorer hypoxia tolerance in the former species. The relative resilience of respiratory, cardiovascular and osmoregulatory processes to salinity and DO variations likely contribute to distinct habitat distributions of the two crab species on New Zealand shorelines, although behaviour and inter-specific interactions may also play important roles. Environmental change, in the form of coastal erosion and anthropogenic contamination of estuaries, has the potential to disturb the delicate niche separation that exists between these species.

## Teaser

Lay summary

In response to acute salinity change or hypoxia, *Hemigrapsus crenulatus* exhibited better maintenance of osmoregulatory and cardiovascular function than *Hemigrapsus sexdentatus*. The greater physiological resilience of *H. crenulatus* is consistent with its habitation of an ecological niche that promotes greater exposure to environmental stressors than that of *H. sexdentatus.*

## Introduction

Intertidal environments are in constant flux. Over the course of a tidal cycle, an aquatic organism living in an intertidal setting may experience changes in water availability, variable water salinity, temperature oscillations and fluctuations in dissolved oxygen (DO). For example, tidal pool DO has been shown to range from 10 to 955 μmol l^−1^ (~0.8–87 kPa) over the course of 6 h (Legrand *et al.,* 2018), whilst salinity in estuaries can range from near zero to hypersaline depending on the magnitude of freshwater influx (Montagna *et al.,* 2018). This instability of environmental physicochemical factors challenges organism homeostasis. Animals that live in these settings must employ physiological strategies that allow them to maintain function despite rapid environmental fluctuation and/or enact behaviours that minimize their exposure to these factors.

Crabs are common inhabitants of intertidal zones and employ a number of physiological mechanisms that enable them to withstand fluctuations in environmental variables such as salinity and DO. Osmoregulating crab species maintain haemolymph osmolality and ion concentrations through the co-ordinated actions of epithelial transporters (Péqueux, 1995). However, epithelial transport may incur a metabolic cost that can be manifested as an increase in oxygen consumption (Shinji *et al.,* 2009). Altered energy demands may also be reflected in changes in cardiovascular parameters. For example, tachycardia is commonly reported in crabs exposed to dilute salinities (Hume and Berlind, 1976; Taylor, 1977; McGaw and McMahon, 1996). This increase in heart rate may facilitate oxygen loading at the gills and thereby fuel the increased metabolic demands of osmoregulation (Taylor, 1977). In conditions of declining DO, bradycardia is frequently reported in crabs (Airriess and McMahon, 1994; McMahon, 2001; McGaw and McMahon, 2003), a response that slows haemolymph flow through the gills, facilitating oxygen transport under conditions where the oxygen uptake gradients are compromised relative to normoxic waters (McMahon, 2001). However, at a certain critical oxygen partial pressure (*P*O_2_), known as the *P*_crit_, regulation fails and the animal transitions from oxyregulation to oxyconformation (Yeager and Ultsch, 1989). This is associated with a greater reliance on anaerobic metabolism. Critically, because of the metabolic costs associated with osmoregulation and the reduced energy provided by anaerobic metabolism, a decrease in DO might ultimately compromise the ability of a crab to maintain extracellular fluid osmolality and ion concentrations (Lucu and Ziegler, 2017).

On New Zealand coastlines, two species of crabs belonging to the genus *Hemigrapsus* are regularly encountered. *Hemigrapsus sexdentatus* (Hilgendorf) (formerly *H. edwardsi*; McLay and Schubart, 2004), the purple rock crab, has a relatively wide ecological niche. It is mainly distributed on stony beaches, occupying the high to mid-tide zone (McLay, 1988). This species does not burrow, but instead may be found sheltering under stones. Notably, *Hemigrapsus sexdentatus* is often associated with freshwater inputs (Williams, 1969) and as a semi-terrestrial species, largely avoids fluctuations in seawater (SW) salinity associated with tidal rhythms. Conversely, *Hemigrapsus crenulatus* (Milne-Edwards), known as the hairy-handed crab, mostly occupies estuaries and lower intertidal zones. It is commonly associated with mud-flats and in these and other soft substrates, it will burrow (McLay, 1988). Sheltering in the burrow during a tidal cycle will expose the crab to a declining DO, whilst riverine inputs at low tide reduce ambient salinity. Both of these crabs are tolerant to variable salinities (Hicks, 1973) and are known to be good osmoregulators, maintaining haemolymph osmolality above SW osmolality in dilute waters (Taylor and Seneviratna, 2005; Lee *et al.,* 2010; Urzúa and Urbina, 2017). To date, however, little is known regarding the tolerance of either of these species to low DO. Given the propensity of *H. crenulatus* to burrow into potentially anoxic, organic-rich, muddy sediments (McLay, 1988), this species would be predicted to be more hypoxia tolerant than *H. sexdentatus*.

The aim of the current study was to investigate the responses of *H. crenulatus* and *H. sexdentatus* to acute changes in DO and salinity, characteristic of those occurring during a tidal cycle. Our hypothesis was that differences in physiological capacity for homeostasis in response to environmental stressors such as low DO and reduced salinity, may contribute towards the distinct ecological niches of these two *Hemigrapsus* species on New Zealand coastlines and may ultimately determine their capacity to withstand anthropogenic pressures on their habitats.

## Materials and methods

### Animal collection and maintenance

Male *H. crenulatus* {mean [±standard error of mean (SEM)] mass = 11 (±2) g} were collected from the Avon-Heathcote Estuary/Ihutai (43°33′S, 172°43′E), whilst male *H. sexdentatus* (33 ± 3 g) were collected from Waipara Beach (43°09′S, 172°48′E), both locations in the Canterbury province of New Zealand. Crabs were immediately transported to the University of Canterbury, where they were held in recirculating natural SW (salinity ~ 35) maintained at 15°C and subjected to a 12-h light:12-h dark photoperiod, for at least a week before experimentation. During acclimation to holding conditions, crabs were fed every other day on fresh mussels, although feeding was withheld 24-h prior to experimentation. All animal procedures were approved by the University of Canterbury Animal Ethics Committee.

### Oxygen consumption and determination of critical *P*O_2_

Oxygen consumption was measured using closed-boxed respirometry, via methods described previously for freshwater crayfish (Broughton *et al.,* 2017). Briefly, a 450-ml Perspex respirometer was placed in a 15°C water bath and the respirometer filled with water at one of five salinities [0.7 (2% SW), 9 (25% SW), 18 (50% SW), 35 (100% SW) or 53 (150% SW)]. Low salinities were made by appropriate dilutions of natural SW collected from Lyttleton Harbour (43°36′S, 172°42′E) with distilled water, whilst the hypersaline water was made by adding artificial sea salt (Instant Ocean) to natural SW. Salinities were confirmed by vapour pressure osmometry (Wescor Vapro). After 10 min of vigorous aeration and circulation of the bathing medium through the respirometer, an individual crab (*n* = 6), was added into a chamber, which was then sealed and measurement of oxygen consumption commenced immediately thereafter (see Discussion). An indwelling oxygen electrode (Strathkelvin) facilitated continuous monitoring of respirometer *P*O_2_ until it reached 1.1 kPa [6% of the initial, fully-oxygenated, water (18.7 kPa); ~12 h]. These data were binned into 5 min intervals and the first 5-min period of each hour was used to plot the decline in *P*O_2_ with time for each individual crab. Oxygen consumption rate (MO_2_; μmol g^−1^ h^−1^) was calculated as follows:

(1) \begin{equation*}{MO}_2=\frac{\left(a\times \Delta{PO}_2\times V\right)}{\left(w\times t\right)}\end{equation*}

where *a* is the oxygen capacitance of water at 15°C (in μmol l^−1^ kPa^−1^), △*P*O_2_ is the change in *P*O_2_ in kPa, *V* is the volume of the respirometer (corrected for the presence of the crab), *w* is the mass of the crab in g and *t* is the time in h. Control respirometers (with no animal) were used to confirm that observed changes in *P*O_2_ were not the result of factors such as the biological oxygen demand of the exposure water or respirometer failure. The relationship between MO_2_ and *P*O_2_ was graphed and a piecewise regression was performed using R statistical software to identify the transition point at which crabs change from oxyregulation to oxyconformation (i.e. the critical *P*O_2_ or *P*_crit_).

### Acute salinity and hypoxia exposures

Individual crabs (*n* = 6 per species per treatment) were added directly to 450-ml Perspex chambers held within a recirculating water bath at 15°C, containing waters that differed either in salinity (0.7, 9, 18, 35 or 53) or DO [in kPa (% oxygen saturation): 1.1 (6), 4.7 (25), 9.3 (50) or 18.7 (100)]. Salinity exposures were conducted with vigorous aeration, whilst DO exposures were conducted in full-strength SW (salinity 35). In the case of DO exposures, water *P*O_2_ was held constant through bubbling of either air or nitrogen gas, monitored via an indwelling DO probe (Hach). Exposures were conducted for 6-h to mimic a tidal interval and measures over this period may therefore reflect a transitional physiological state (see Discussion). Two sets of six crabs were used, one set for determination of heart rate and the other set for haemolymph sampling, which was thereafter analysed for osmolality and ions (sodium, potassium and chloride).

Heart rate was measured continuously using an infrared sensor attached non-invasively to the carapace, as described in Broughton *et al.* (2017). The attachment of the sensor housing was performed 2–3 days prior to experimentation. The mean heart rate over a 5 min period at the end of each hour of the exposure was recorded, excluding any intervals where the heart was not beating.

At the conclusion of the 6-h exposure, haemolymph was extracted from the infrabranchial sinus at the base of the walking legs. Haemolymph osmolality was determined using a vapour pressure osmometer (Wescor), sodium and potassium were assessed via flame photometry (Sherwood), whilst chloride was measured using a digital chloridometer (Labconco).

### Statistical analyses

The two crab species differed significantly in mass (*H. crenulatus* = 11 g; *H. sexdentatus* = 33 g). Consequently, to avoid complications associated with mass differences, the responses of each crab species to salinity and DO were assessed independently (i.e. via one-way ANOVA) to examine the effects of experimental variables (i.e. DO, salinity) within a species. An initial two-way ANOVA showed that the heart rate of both species was not dependent on time (i.e. did not vary over the 6 h of exposure). Subsequently, one-way ANOVAs were conducted using the mean heart rate across the 6-h exposure for each individual.

For all analyses, tests of normality (Kolmogorov-Smirnov) and equality of variance (Levene’s) were first conducted. When data were confirmed as parametric a one-way ANOVA was performed, followed by a *post hoc* Tukey’s test. Non-parametric data were either log-transformed (effect of DO on *H. sexdentatus* heart rate) and then subjected to parametric ANOVA or interrogated via a non-parametric Kruskal-Wallis ANOVA, followed by a Dunn’s post hoc test (effect of DO on *H. crenulatus* haemolymph potassium and chloride). For all analyses, an alpha value of 0.05 was considered significant. Throughout the manuscript, values are reported as means ± SEM.

## Results

**Figure 1:**
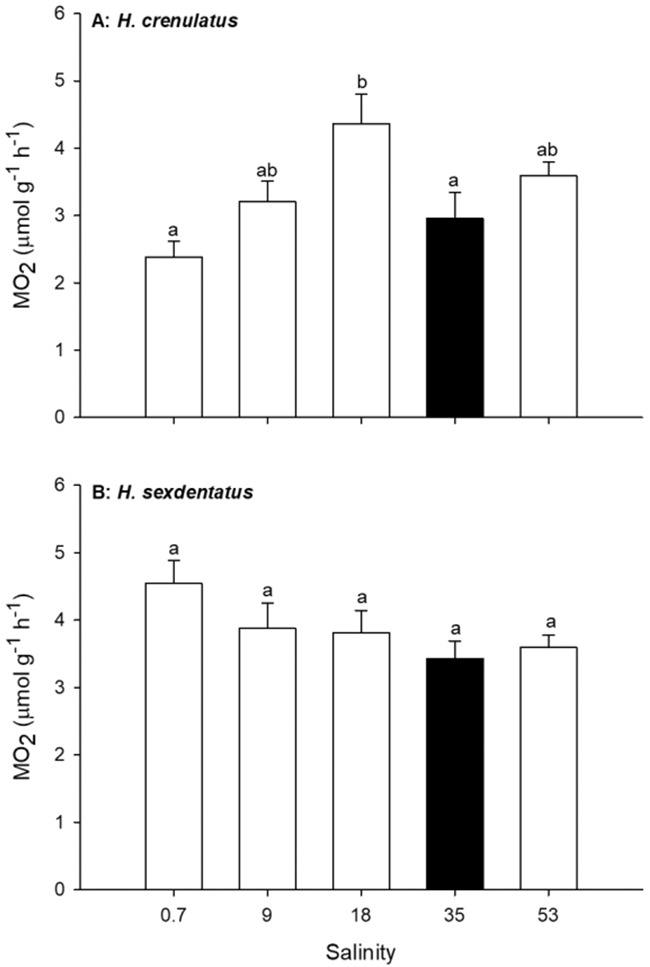
Effect of exposure salinity on normoxic MO_2_ in *H. crenulatus* (A) and *H. sexdentatus* (B). Plotted values represent means (±SEM) of six replicates. Within panels, bars sharing lowercase letters are not significantly different (one-way ANOVA, *α* = 0.05).

The normoxic MO_2_ of *H. crenulatus* varied significantly as a function of exposure salinity (one-way ANOVA, *P* = 0.004; Fig. 1A). A fall in exposure salinity from full-strength SW (35) to 50% SW (18) resulted in a significant 1.5-fold increase in MO_2_. At lower salinities and in the hypersaline test condition, the MO_2_ of *H. crenulatus* was unchanged relative to the control. This pattern was distinct from that observed for *H. sexdentatus* (Fig. 1B). In this species, there were no significant effects of salinity on MO_2_, although a trend towards increasing MO_2_ with decreasing salinity could be observed (one-way ANOVA, *P* = 0.132).

**Figure 2:**
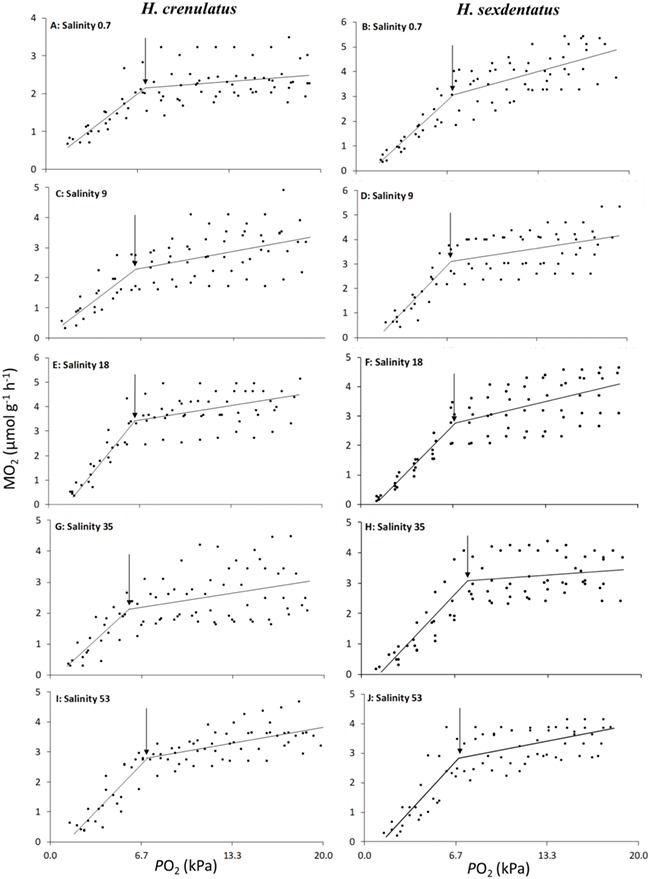
Effect of salinity [0.7 (A and B), 9 (C and D), 18 (E and F), 35 (G and H), 53 (I and J)] on the relationship between water *P*O_2_ and MO_2_ in *H. crenulatus* and *H. sexdentatus*. Plotted values represent all data for six crabs, with lines fitted and breakpoints (i.e. *P*_crit_) derived via a piecewise regression performed using R statistical software

Closed-box respirometry resulted in a decline in *P*O_2_ as the crab consumed the oxygen in the respirometer (Fig. 2). Plotted values in this figure represent datapoints for all individual crabs within a given stressor level. In both crab species, in all water salinities, MO_2_ remained relatively constant until a water *P*O_2_ of ~5.5–7.5 kPa. Thereafter, the capacity of the crab to regulate oxygen consumption failed and a rapid decline in MO_2_ with falling water *P*O_2_ was observed. Using values calculated for each individual and then averaged, the *P*_crit_ was determined to be 5.9 ± 0.9 kPa for *H. crenulatus* in full-strength SW (i.e. salinity = 35). Under the same exposure conditions, the mean calculated *P*_crit_ for *H. sexdentatus* was 7.6 ± 0.2 kPa. Values of *P*_crit_ did not differ as a function of exposure water salinity for either species (one way ANOVAs; *P* = 0.37 for *H. crenulatus*, *P* = 0.08 for *H. sexdentatus*; data not shown).

**Figure 3:**
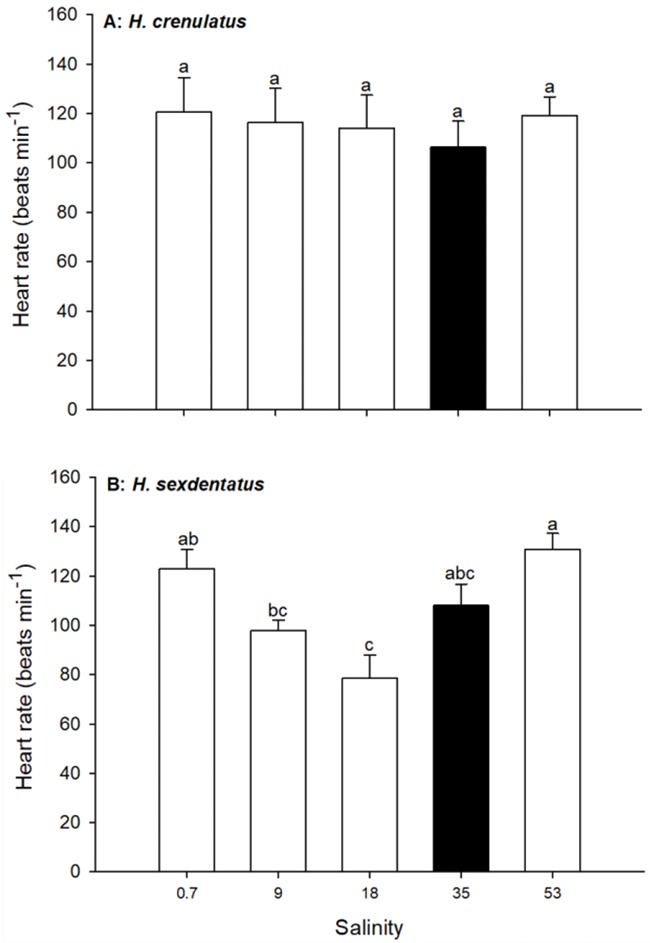
Effect of exposure salinity on heart rate in *H. crenulatus* (A) and *H. sexdentatus* (B). Plotted values represent means (±SEM) of six replicates. Within panels, bars sharing lowercase letters are not significantly different (one-way ANOVA, *α* = 0.05).

During a 6-h exposure to a range of water salinities, the heart rate of *H. crenulatus* remained unchanged (one-way ANOVA, *P* = 0.92; Fig. 3A), ranging from 106 ± 11 to 121 ± 14 beats min^−1^ for salinities of 35 and 0.7, respectively. Conversely, there was a significant effect of salinity on the mean heart rate of *H. sexdentatus* (one-way ANOVA, *P* < 0.001; Fig. 3B). The mean heart rate of 79 ± 9 beats min^−1^ at a salinity of 18 (50% SW) was significantly lower than that at the two salinity extremes [0.7 (2% SW) 123 ± 8 beats min^−1^; 53 (150% SW), 131 ± 7 beats min^−1^]. There were, however, no significant differences in *H. sexdentatus* heart rate in any salinity relative to the full-strength SW control.

**Figure 4:**
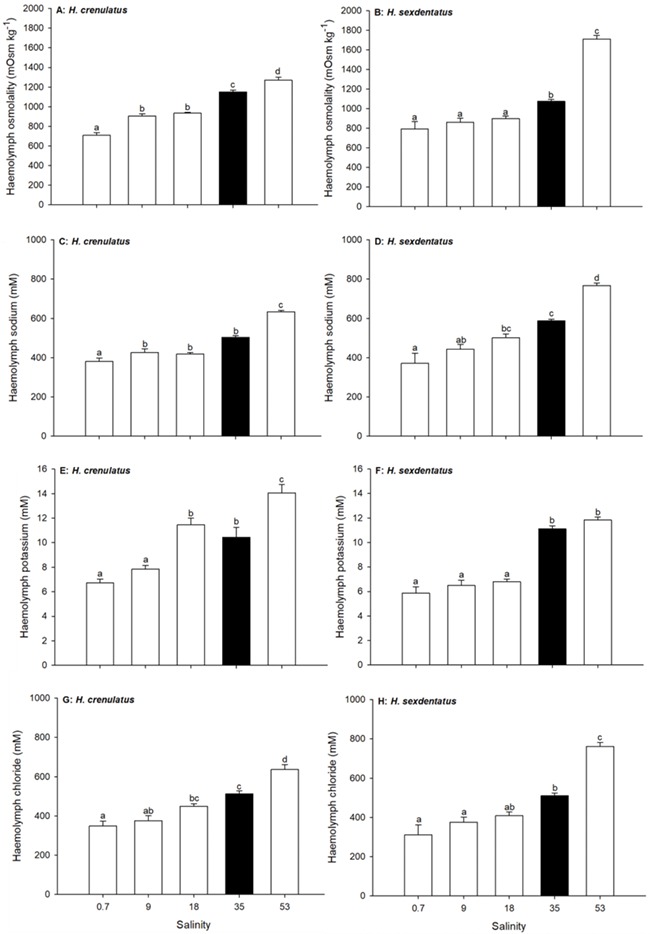
Effect of exposure salinity on haemolymph osmolality (A and B), sodium (C and D), potassium (E and F) and chloride (G and H) ion concentrations in *H. crenulatus* (A, C, E and G) and *H. sexdentatus* (B, D, F and H). Plotted values represent means (±SEM) of six replicates. Within panels, bars sharing lowercase letters are not significantly different (one-way ANOVA, *α* = 0.05).

Analysis of haemolymph osmolality showed that both crabs were hyperosmotic regulators in dilute salinities (Fig. 4A and B). In both *H. crenulatus* and *H. sexdentatus* haemolymph osmolality dropped significantly in crabs exposed for 6-h to a salinity of 18 (50% SW) relative to crabs maintained at a salinity of 35 (100% SW; overall one way ANOVA, *P* < 0.001 for both species). Thereafter, further dilution of the exposure medium had no further significant effect on the osmolality of *H. sexdentatus* haemolymph, whereas at a salinity of 0.7 (2% SW), *H. crenulatus* osmolality was significantly lower than that at all other salinities. In hypersaline conditions (salinity = 53; 150% SW), both species displayed a significant increase in haemolymph osmolality.

Patterns for haemolymph ions were generally similar to those for osmolality (Fig. 4C–H). Relative to the control (salinity 35, 100% SW), crabs of both species exposed to dilute salinities displayed lower haemolymph ion concentrations, whereas crabs exposed to higher salinities displayed elevated haemolymph ion concentrations (one way ANOVAs, all *P* < 0.001). The threshold salinities, at which ion concentrations became significantly different, varied between ions and species. For sodium, potassium and chloride in *H. crenulatus*, statistically significant differences relative to the control were observed in crabs exposed to salinities of 0.7, 9 and 9, respectively (Fig. 3C, E and G). For sodium, potassium and chloride in *H. sexdentatus*, statistically significant differences relative to the control were observed in the haemolymph of crabs exposed to salinities of 9, 18 and 9, respectively (Fig. 4D, F and H). The single exception where an increase in exposure salinity to 53 (150% SW) did not lead to a statistically increased haemolymph ion concentration was for chloride in *H. sexdentatus*.

**Figure 5:**
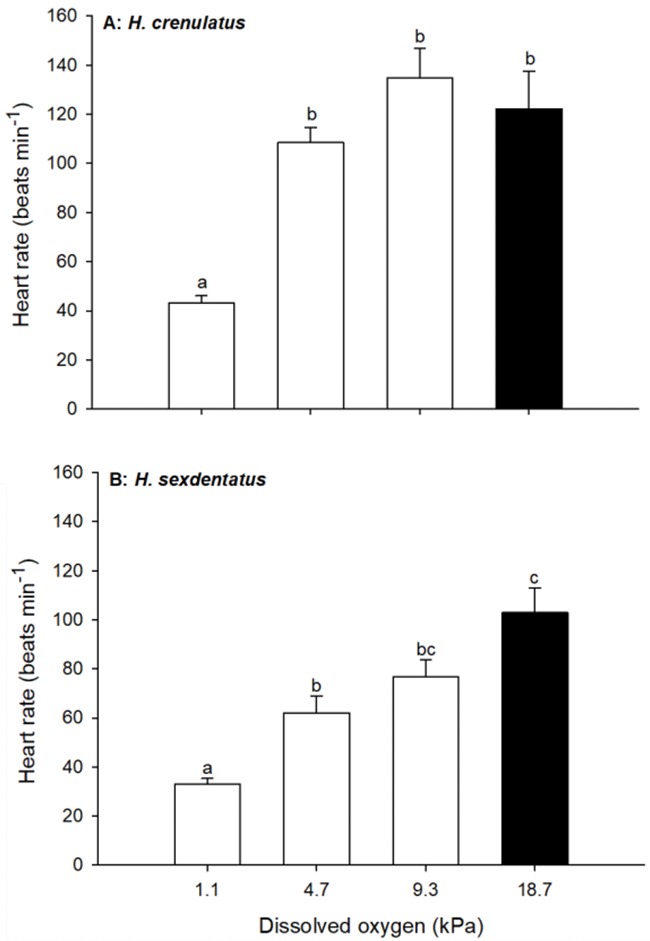
Effect of exposure *P*O_2_ on heart rate in *H. crenulatus* (A) and *H. sexdentatus* (B). Plotted values represent means (±SEM) of six replicates. Within panels, bars sharing lowercase letters are not significantly different (one-way ANOVA, *α* = 0.05).

A 6-h exposure to lowered water DO had a significant overall effect on heart rate for both *H. crenulatus* (one-way ANOVA, *P* < 0.001; Fig. 5A) and *H. sexdentatus* (one-way ANOVA, *P* < 0.001; Fig. 5B). In the former species, heart rate was statistically unchanged relatively to the normoxic control (18.7 kPa), until exposure to 1.1 kPa. In crabs from this treatment, a mean heart rate of 43 ± 3 beats min^−1^ was observed, a value just 35% of that measured in normoxic waters (122 ± 15 beats min^−1^). For *H. sexdentatus*, a significant fall in heart rate with declining DO was observed at 4.7 kPa, a treatment of higher *P*O_2_ than that observed to have the same effect in *H. crenulatus*. In the lowest *P*O_2_ (1.1 kPa), the heart rate of *H. sexdentatus* was 33 ± 2 beats min^−1^, a value 32% of that recorded in normoxic crabs of this species (103 ± 10 beats min^−1^). 

**Figure 6:**
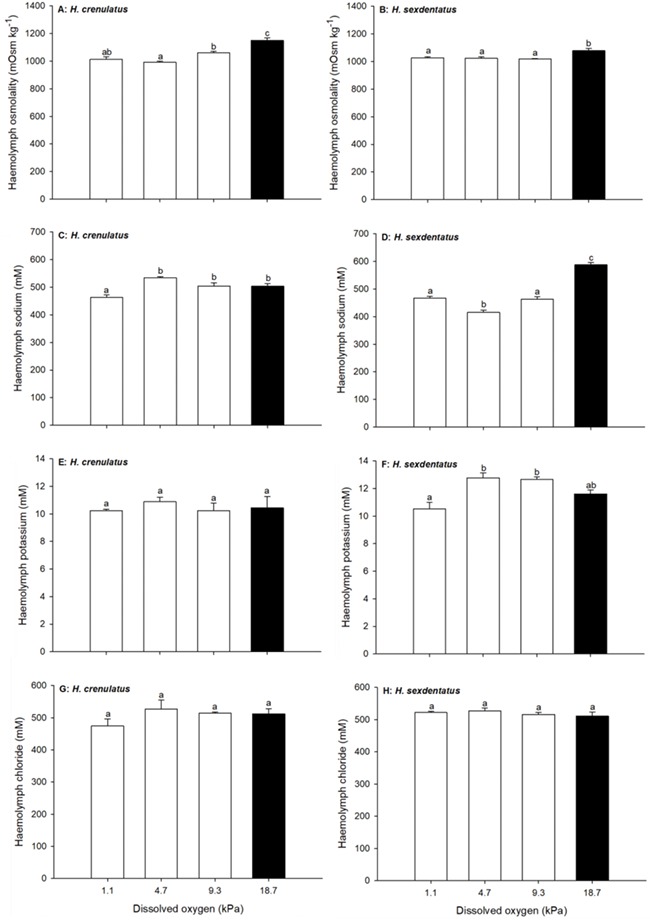
Effect of exposure *P*O_2_ on haemolymph osmolality (A and B), sodium (C and D), potassium (E and F) and chloride (G and H) ion concentrations in *H. crenulatus* (A, C, E and G) and *H. sexdentatus* (B, D, F and H). Plotted values represent means (±SEM) of six replicates. Within panels, bars sharing lowercase letters are not significantly different (all one-way ANOVA, except *H. crenulatus* potassium and chloride which were assessed via Kruskal-Wallis ANOVA; *α* = 0.05).

A 6-h exposure to reduced *P*O_2_ resulted in decreases in haemolymph osmolality in both crab species (Fig. 6A and B; one way ANOVAs; *P* < 0.001 for *H. crenulatus*; *P* = 0.002 for *H. sexdentatus*). All treatments displayed osmolality values that were lower than the normoxic control for both species. Haemolymph sodium concentrations also differed significantly as a function of exposure *P*O_2_ (one way ANOVAs, both *P* < 0.001; Fig. 6C and D). For *H. crenulatus,* haemolymph sodium was maintained at normoxic control levels, until the lowest tested exposure *P*O_2_ (1.1 kPa), where the value of 463 ± 10 mM was significantly distinct from crabs in all other waters (Fig. 6C). Conversely, in *H. sexdentatus* dropping the *P*O_2_ from 18.7 to 9.3 kPa caused a significant 22% fall in haemolymph sodium (Fig. 6D). A further significant decline in *H. sexdentatus* haemolymph sodium was noted at 4.7 kPa. At 1.1 kPa, there was a small but significant increase, in haemolymph sodium relative to the 4.7 kPa treatment, but this value was still significantly reduced with respect to the control. Relative to normoxia, there were no significant effects of *P*O_2_ on haemolymph potassium (Fig. 6E and F) or haemolymph chloride (Fig. 6G and H), for either crab species. However, for *H. sexdentatus*, crabs from the 1.1 kPa exposure condition displayed haemolymph potassium values that were significantly reduced relative to the 4.7 and 9.3 kPa treatments.

## Discussion

### Respiratory responses to acute salinity change

The MO_2_ of *H. sexdentatus* acutely exposed to either hypo- or hyper-saline waters was unchanged across all tested salinities (Fig. 1B). In contrast, an increase in MO_2_ was observed in *H. crenulatus* as crabs moved from full-strength SW to 50% SW (salinity = 18; Fig. 1A). In other crab species, increases in MO_2_ with declining salinity are commonly reported (Taylor *et al.,* 1977b; Shumway, 1983; Normant and Gibowicz, 2008), although some authors have reported MO_2_ increases in hypersaline waters (Ramaglia *et al.,* 2018) or a lack of change in MO_2_ with salinity (Winch and Hodgson, 2007; Theuerkauff *et al.,* 2018). Differences between studies are likely due to diverse experimental protocols (most notably whether the measurement occurs after acute exposure or after acclimation) and differences in the osmoregulatory capacity of the test species.

However, the current findings of a transient effect or lack of response of MO_2_ to salinity are distinct from previous studies in *Hemigrapsus*. For example, employing a protocol comparable to that of the current work, an acute (2–6 h) salinity exposure in *H. takanoi* resulted in a higher MO_2_ in lower salinities (Shinji *et al.,* 2009). We propose that differences in experimental outcomes reflect subtle differences in ecological niches, which are known to vary between *Hemigrapsus* species and between populations of the same species from different regions (e.g. McLay, 1988).

Over the course of a 6-h acute exposure neither crab species displayed changes in *P*_crit_. The *P*_crit_ is widely used as an indicator of hypoxia tolerance in aquatic biota, representing the point at which an oxyregulator can no longer maintain metabolic rate and thereafter oxygen consumption declines as a function of declining *P*O_2_ (Yeager and Ultsch, 1989). Previous studies have shown that exposure of crabs (*Carcinus maenas* and *Carcinus aestuarii*) to dilute salinities leads to an increase in *P*_crit_ (Taylor *et al.,* 1977a; Rivera-Ingraham *et al.,* 2016). This is likely a consequence of the greater oxygen demands of crabs in lower salinities in these studies, such that the onset of the transition between aerobic and anaerobic metabolism occurs at a higher *P*O_2_. Given that the crabs in the current study did not show a consistent pattern of increased metabolic costs in dilute salinities, then the finding of a salinity-independent *P*_crit_ is not surprising.

### Cardiovascular responses to acute salinity change

Exposure of crabs to salinity change is known to induce tachycardia (Hume and Berlind, 1976; Taylor, 1977; McGaw and McMahon, 1996), although in some osmoconforming species bradycardia may be observed (McGaw, 2006). However, in the current study, a change in exposure salinity had no impact on heart rate relative to controls where heart rates were monitored in full-strength SW. Tachycardia in response to salinity change has been proposed as a mechanism that facilitates oxygen uptake, thereby fuelling enhanced metabolic costs associated with osmoregulation and/or increased locomotor costs (Taylor, 1977). Consequently, our data showing an absence of tachycardia are consistent with our results showing a lack of consistent effect of salinity on MO_2_. Heart rate alone, however, does not always provide a complete picture of cardiovascular change. In crabs, cardiac output can change independently of heart rate due to altered stroke volume, such that heart rate may not accurately reflect changes in cardiovascular dynamics (McGaw and McMahon, 2003). It is therefore possible that although heart rates were relatively consistent across different salinities, cardiac output may not have been. However, even if changes in cardiovascular physiology occurred, it seems as though these acted to maintain MO_2_ rather than to meet increased costs associated with hypo- or hyper-saline exposure.

### Osmotic and ionic responses to acute salinity change

Both *H. crenulatus* and *H. sexdentatus* maintained haemolymph osmolality and ion concentrations below and above, those of more dilute or concentrated salinities, respectively. Although the current study only determined osmolality after 6 h (i.e. a tidal cycle) and it can take up to 48 h for osmoregulatory status to develop completely (Lovett *et al.,* 2001), our findings were consistent with previous work on these species (Taylor and Seneviratna, 2005; Lee *et al.,* 2010; Urzúa and Urbina, 2017).

In both crabs, haemolymph osmolality dropped significantly as animals acclimated to full-strength SW were placed in 50% SW (salinity = 18; Fig. 4A and B). The effects of salinity on haemolymph ions were more distinct and highlight *H. crenulatus* as the stronger ion regulator. For example, the threshold salinity at which haemolymph sodium ion concentration differed significantly from the control was 0.7 and 9, for *H. crenulatus* and *H. sexdentatus*, respectively. For potassium ion, the salinity threshold was 9 for *H. crenulatus* and 18 for *H. sexdentatus.* Distinct patterns for haemolymph osmolality and haemolymph ions within the same treatment group are likely due to changes in unmeasured osmolytes (e.g. amino acids), which are known to fluctuate with external salinity (Findley and Stickle, 1978).

The finding that *H. crenulatus* is the better regulator over the course of a 6-h exposure is generally consistent with previous data regarding the tolerance of these two species to salinity change. For example, Hicks (1973) noted that *H. crenulatus* was more tolerant to dilute salinities than *H. sexdentatus* at an exposure temperature of 15°C. Conversely, Taylor and Seneviratna (2005), found that *H. crenulatus* was less tolerant to low salinities, although this study was conducted on early life-stages. This finding does, however, have some support from the data in the current work. At the lowest tested salinity (0.7), *H. sexdentatus* was able to maintain osmolality at a level statistically indistinct from that in 50% SW (18), whereas in 0.7 salinity water *H. crenulatus* osmolality was statistically lower than that of crabs at 50% SW. This ability to regulate in very dilute salinities has been attributed to the association of this species with freshwater inputs (Williams, 1969). 

In the current work, the mass of the adult crabs differed markedly, with *H. sexdentatus* being three times larger than *H. crenulatus*. A recent study examining the capacity of *H. crenulatus* to regulate haemolymph sodium in response to decreasing salinity noted that larger crabs were better regulators (Urzúa and Urbina, 2017). It is therefore noteworthy that on the basis of mass differences alone, *H. sexdentatus* would be predicted as the better regulator. This indicates that the effects seen in the current study were not simply a consequence of body mass and that a more distinct separation of the osmoregulatory and ionoregulatory capacities of the two species may have been identified if experimental body sizes were equivalent.

### Respiratory responses to hypoxia

Under conditions where crabs were exposed to declining *P*O_2_, resulting from their consumption of oxygen within a sealed chamber, both species initially oxyregulated. This pattern was then superseded by an oxyconforming response once *P*_crit_ was reached (Fig. 2). The measured *P*_crit_ values ranged from 5.5 to 7.6 kPa, in line with previous studies that have characterized *P*_crit_ in intertidal crab species. For example, in *Carcinus* a *P*_crit_ of 5.3 kPa was determined (Taylor, 1981), albeit at a slightly lower experimental temperature than that used in the current study (10 vs. 15°C). In general, intertidal crabs display *P*_crit_ values that are intermediate to the higher values measured in crustaceans that function in well-oxygenated subtidal environments (e.g. 4–11 kPa) and to the lower values determined for species that inhabit poorly oxygenated burrows (1.3–6.7 kPa; Whiteley and Taylor, 2015). Contrary to prediction, the more fossorial species, *H. crenulatus*, did not display a lower *P*_crit_, indicative of higher hypoxia tolerance. Given that differences in cardiovascular responses to hypoxia were discerned in the current work, this suggests that the *P*_crit_ may not be a useful indicator of relative hypoxia tolerance in these species.

### Cardiovascular responses to acute hypoxia

In response to declining *P*O_2_, both *Hemigrapsus* species displayed bradycardia. This is a commonly observed response to hypoxia in crabs (Bradford and Taylor, 1982; Airriess and McMahon, 1994) and is one that is usually accompanied by an increase in stroke volume (McGaw and McMahon, 2003). Together, these changes are thought to aid oxygen loading at the gills by increasing the volume of haemolymph oxygenated and its branchial residence time, whilst also facilitating oxygen unloading at the tissues (McMahon, 2001).

Although both species in the current study displayed bradycardia, the onset of this response differed. For *H. crenulatus*, a significant bradycardia only occurred once *P*O_2_ reached 1.1 kPa, whereas the equivalent value for *H. sexdentatus* was 4.7 kPa. This suggests that *H. sexdentatus* is less hypoxia tolerant. In general, there is a correlation between bradycardia and hypoxia tolerance in crustaceans. In species such as *Cancer pagurus*, a relatively hypoxia-sensitive crab, heart rate decreases as *P*O_2_ declines (Bradford and Taylor, 1982). However, burrowing crustaceans are highly hypoxia-tolerant and often do not exhibit a bradycardia in waters of low *P*O_2_, instead relying on adaptations such as respiratory pigments with high oxygen affinity to maintain metabolic rate (Whiteley and Taylor, 2015).

### Osmoregulatory responses to acute hypoxia

In both *H. crenulatus* and *H. sexdentatus*, exposure to reduced *P*O_2_ resulted in decreases in haemolymph osmolality and sodium ion concentrations (Fig. 6A–D). In the freshwater prawn, a similar effect of hypoxia has been noted (Cheng *et al.,* 2003). This was attributed to haemolymph dilution, which resulted from enhanced water influx associated with elevated ventilation rates. An alternate explanation is that the observed effects are the consequences of osmorespiratory compromise at the gill. A standard response to hypoxia in crabs is to increase ventilation rate, compensating for the reduced *P*O_2_ by bringing larger volumes of water in contact with the gill epithelia (McGaw and McMahon, 1996). This would exacerbate diffusive exchange of ions should any small differences in extracellular and environmental ion concentrations exist. Similarly, the reduction in MO_2_ as *P*O_2_ drops means that there is reduced energy available to restore osmotic balance (Lucu and Ziegler, 2017), which could also result in changes in haemolymph osmolality and ion concentrations.

The threshold of effects of *P*O_2_ on haemolymph osmolality was identical in *H. crenulatus* and *H. sexdentatus*. The drop in *P*O_2_ from 18.7 to 9.3 kPa, induced significant osmolality declines in both species. However, with respect to effects on sodium ion, *H. crenulatus* was better able to maintain concentrations (until a *P*O_2_ of 1.1 kPa), relative to *H. sexdentatus* (statistically significant drop in haemolymph sodium relative to normoxic control at 9.3 kPa). The mechanism for this is unknown, but if *H. crenulatus* had a superior anaerobic capacity to *H. sexdentatus*, then this could provide the energy to better sustain ion regulation over the duration of the short-term 6-h exposure. These data nevertheless suggest that *H. crenulatus* is the more tolerant of the two species to hypoxia, consistent with cardiovascular data and its habitation of environments with greater risk of exposure to low *P*O_2_.

### Methodological and environmental considerations

In the current study, crabs were added into exposure chambers and immediately subjected to physiological investigation. This differs from approaches where the animals are acclimated to the chambers for several hours prior to medium manipulation (e.g. Broughton *et al.,* 2017). Consequently, the responses measured may reflect stress associated with handling. However, analysis of continuous recordings in respirometry experiments showed that any initial elevations in oxygen consumption lasted less than 20 min (data not shown). Some authors have also utilized pauses in heart rate as an indicator of reduced stress in crabs (McGaw and Nancollas, 2017). In our study, the mean time of acardia onset in control crabs was 86 min (data not shown), suggesting that crabs settled relatively quickly into chambers. Nevertheless, we cannot rule out that a component of time-integrated measurements (i.e. haemolymph osmolality and ions) could reflect handling stress.

The ability of our study to draw conclusions regarding the importance of physiology in shaping niche habitation is also limited by a lack of knowledge of the physical and chemical variability in the habitats, and more specifically the microhabitats, of the study species. For example, it is possible that the tested levels of salinity and DO are beyond the scope of those experienced by the crabs in natural settings. Consequently, interpreting physiological tolerance at physicochemical extremes in the laboratory may be misleading if environmental values for salinity and DO fluctuate over narrower ranges. Furthermore, laboratory studies involve the maintenance of animals under controlled conditions. Given that crab species exhibit physiological responses that are entrained by environmental cues (e.g. McGaw and McMahon, 1998), then this represents an additional challenge when attempting to ascribe ecological niche habitation to physiological tolerance.

## Conclusion

*H. crenulatus* appeared to be more tolerant to low environmental *P*O_2_ than *H. sexdentatus*, befitting its habitation of environments where hypoxia may occur (i.e. burrows in muddy substrates; McLay, 1988). Similarly, lower onsets of salinity effects on haemolymph sodium and potassium ion concentrations in *H. crenulatus* relative to *H. sexdentatus* hint at a greater short-term tolerance of the former species to dilute salinities, at least in the 9–18 (25–50% SW) range. This would be consistent with a crab that inhabits the lower intertidal zone, where exposure to salinity fluctuations is more commonly encountered.

Overall, however, the effects of DO and salinity on *Hemigrapsus* physiology were relatively minor, suggesting that factors other than physiology will contribute to the distinct niches of these two species along New Zealand coastlines. For example, the current study exposed crabs under conditions where normal behavioural responses could not be enacted. The importance of responses such as emersion and avoidance in crabs exposed to environmental stressors is well-established (McGaw *et al.,* 1999; Bell *et al.,* 2009) and will be of relevance in determining habitat selection in natural settings. Inter-specific interactions will also play a role. Indeed, it is notable that in Chile *H. crenulatus* is the sole *Hemigrapsus* species and occupies a wider and more exposed environmental niche than it does in New Zealand (McLay *et al.,* 2011). It is also important to note that the current study only examined salinity and DO as stressors. Similar studies of sympatric intertidal crab species have identified temperature and tolerance to this stressor, as the major factor explaining differences in species distributions (Jensen and Armstrong, 1991; Stillman and Somero, 1996).

Finally, the current study has implications for conservation of these species. The coastal habitats of *Hemigrapsus* in the Canterbury region of New Zealand are subjected to anthropogenic change. For example, the Avon-Heathcote Estuary/Ihutai receives nutrient inputs that result in water quality metrics that exceed regulatory trigger values and which lead to extensive anoxia (Bolton-Ritchie and Main, 2005). Beach habitats are exposed to coastal erosion, which will worsen with predicted sea level rise (MfE, 2017). This can result in changes in tidal profiles and narrowing of ecological niches. This is exacerbated by modifications of coastal infrastructure such as sea walls, which may further condense optimal habitat and expose animals to more severe fluctuations in environment (Gittman *et al.,* 2016). The current work indicates that the *Hemigrapsus* crab species found on New Zealand coasts have sufficient physiological plasticity to enable them to adjust niche in response to environmental change. However, shifts in ecological niches may increase inter-specific competition and ultimately could lead to exclusion of the less robust species.
